# Simulation Analysis of Temperature Change in FDM Process Based on ANSYS APDL and Birth–Death Element Technology

**DOI:** 10.3390/mi16101181

**Published:** 2025-10-19

**Authors:** Yuehua Mi, Seyed Hamed Hashemi Sohi

**Affiliations:** 1 School of Mechanical Manufacturing and Energy Engineering, School of Graduate Studies, Mapúa University, Manila 1002, Philippines; shhashemisohi@mapua.edu.ph; 2 School of Mechanical and Electrical Engineering, Zhengzhou Business University, Zhengzhou 451200, China; 3 Zhengzhou Intelligent Electromechanical Engineering Research Center, Zhengzhou 451200, China

**Keywords:** Fused Deposition Modeling (FDM), ANSYS APDL, birth–death element technology, thermomechanical coupling, stress and strain

## Abstract

During the Fused Deposition Modeling (FDM) molding process, temperature changes are nonlinear and instantaneous, which is a key parameter affecting FDM printing efficiency, molding accuracy, warpage deformation, and other factors. This study presents a finite element simulation framework that integrates ANSYS Parametric Design Language (APDL) with birth–death element technology to investigate the temperature evolution and thermomechanical behavior during the FDM process. The framework enables dynamic simulation of the complete printing and cooling cycle, capturing the layer-by-layer material deposition and subsequent thermal history. Results indicate that temperature distribution follows a gradient pattern along the printing path, with rapid heat dissipation at the periphery and heat accumulation in the central regions. Thermomechanical coupling analysis reveals significant stress concentration at the part bottom (310 MPa) and progressive strain increase from bottom (3.68 × 10^−5^ m) to top (2.95 × 10^−4^ m). Experimental validation demonstrates strong agreement with numerical predictions, showing maximum temperature deviations below 8% and strain distribution errors within 5%. This integrated approach provides an effective tool for predicting thermal-induced deformations and optimizing FDM process parameters to enhance part quality.

## 1. Introduction

Fused Deposition Modeling (FDM) has emerged as a prominent additive manufacturing technology that leverages continuous high-temperature heating to melt thermoplastic filaments. These molten filaments are extruded through a precision nozzle and deposited layer-by-layer to construct three-dimensional structures, enabling the rapid fabrication of complex geometries [1]. Owing to its inherent advantages, including cost-effectiveness, high production efficiency, and operational simplicity, FDM has been widely adopted across diverse industrial sectors, such as biomedical device manufacturing (e.g., biomedical-grade biodegradable polylactic acid (PLA) scaffolds [2]), automotive lightweight component production [3], and customized consumer product development [4]. However, the dynamic nature of filament deposition in FDM introduces a critical challenge: maintaining the filament in a stable molten state is essential for ensuring robust interlayer bonding, and temperature has been identified as the pivotal parameter directly dictating printing accuracy, mechanical properties of printed parts, and post-printing warpage [5,6].

To address these temperature-related challenges, finite element simulation has evolved into a dominant approach for investigating temperature field evolution in FDM [7,8]. Recent studies have focused on three key directions: elucidating the correlation between temperature variation and part-forming quality, uncovering the fundamental laws governing temperature distribution, and optimizing process parameters to mitigate thermal defects [9,10]. For instance, Mosleh et al. [11] employed COMSOL Multiphysics to simulate fluid flow and heat transfer in FDM-printed wire harnesses, demonstrating that increasing nozzle temperature or layer thickness elevates the temperature in the vicinity of the nozzle—whereas for thin filaments, platform temperature exerts a dominant influence on the overall layer temperature. Nevertheless, their static modeling framework failed to capture the dynamic “material accumulation effect” intrinsic to FDM, resulting in notable discrepancies between simulated and experimental interlayer temperature gradients. In a related study, Baeza-Campuzano et al. [12] analyzed six printing speeds under a fixed nozzle temperature of 230 °C, observing that higher printing speeds induce severe temperature fluctuations and confirming the regulatory role of filament viscosity on nozzle wall shear stress. However, their simulation neglected the transient activation of newly deposited material elements, leading to overestimated cooling rates for the initial 5–10 layers [13].

Other scholars have explored specialized simulation approaches to address these limitations. Apaolu Turan et al. [14] developed custom simulation code to investigate interlayer and intralayer reheating effects, parameter sensitivity, and realistic printing trajectories, yielding valuable insights into temperature–structure interactions. Unfortunately, this code lacked the numerical stability and computational efficiency of commercial FE platforms (e.g., ANSYS), leading to convergence issues in large-scale models (e.g., parts with more than 10,000 elements [15]). Schmidtke et al. [16] focused on temperature control, proposing a model predictive control (MPC) scheme for 3D printing that accounts for process constraints and arbitrary temperature references, using partial differential equations (PDEs) to regulate spatial temperature distribution. However, this work prioritized control algorithm design over the physical mechanism of heat diffusion during layer-by-layer deposition, limiting its applicability to fundamental analyses of the FDM temperature field [17].

Experimental studies have also highlighted gaps in thermomechanical coupling understanding. Zniker et al. [18] investigated the effect of printing temperature (320–350 °C) on the mechanical properties and failure behavior of FDM-printed polyphenylene sulfide (PPS) parts via impact tests, revealing that elevated temperatures enhance toughness but exacerbate post-printing shrinkage. However, their supporting simulation excluded thermomechanical coupling effects, precluding the establishment of a quantitative link between temperature gradients and structural deformation [19]. Similarly, Marizky et al. [20] measured the shrinkage of PLA parts at nozzle temperatures of 210 °C, 220 °C, and 230 °C, finding that the shrinkage rate of tensile and bending specimens reached a maximum at 230 °C. However, their model assumed constant material stiffness, contradicting the well-documented temperature-dependent softening behavior of PLA (e.g., its elastic modulus decreases by 40% when heated from 25 °C to 150 °C [21]).

In summary, temperature variation is a core factor influencing FDM printing efficiency, part-forming accuracy, and warpage [22,23]. While existing studies have explored temperature-related issues from perspectives such as filament melting dynamics, nozzle temperature optimization, and process parameter tuning [24,25], three critical research gaps persist. First, few simulations capture the full-cycle temperature evolution across the entire FDM process (from filament deposition to post-printing cooling); most are confined to single-stage analysis (e.g., extrusion only or cooling only [26]). Second, thermomechanical coupling analysis—an essential tool for revealing how temperature variation drives structural deformation—is rarely integrated into FE simulations of the FDM temperature field [27,28]. Third, traditional simulation methods lack flexibility and efficiency: fixed-platform modeling software (e.g., COMSOL Multiphysics 5.3a) cannot adapt to dynamic process adjustments, while self-developed codes suffer from low computational efficiency and poor numerical stability.

To overcome these limitations, this research develops an integrated computational framework combining ANSYS APDL with birth–death element technology to simulate the complete FDM thermal cycle from deposition to cooling. This integration provides three significant advancements: (1) APDL enables automated script generation and parametric adjustment of critical process variables (material properties, heat source characteristics, printing path), overcoming the inflexibility of commercial GUI-based platforms [29]; (2) The birth–death element technique dynamically activates newly deposited material elements while maintaining previously deposited layers, accurately replicating the sequential nature of FDM and addressing the material accumulation effect neglected in previous studies; (3) A synchronized multiphysics data framework establishes quantitative relationships between temperature history and residual stress development [30].

This study aims to: (1) characterize spatiotemporal temperature evolution during FDM printing; (2) analyze temperature variations under thermomechanical coupling conditions; and (3) correlate temperature changes with stress–strain distributions in printed parts. The findings provide theoretical foundations for optimizing FDM process parameters and improving part quality.

## 2. Methodology

### 2.1. Programmable Control of the FDM Process

#### 2.1.1. Ansys Parametric Design Language

Ansys parametric design language (APDL), the scripting interface for ANSYS finite element analysis software, enables users to programmatically develop and optimize models, as well as adjust simulation parameters according to specific requirements [31]. Offering extensive parameterization capabilities, APDL facilitates the definition of parameters, material properties, boundary conditions, loading configurations, solution procedures, and post-processing operations. This allows for the execution of complex simulations beyond standard settings. By employing APDL scripting, precise control over each modeling step and mesh element can be achieved, thereby optimizing the simulation process and enhancing both computational accuracy and efficiency.

#### 2.1.2. Birth and Death Unit Technology

The “birth and death” element technique in ANSYS is a discrete modeling method that controls the activation state of elements. An element is “alive” when activated, in which case it is included in the simulation calculation; conversely, an element set as “dead” is excluded from the calculation and exerts negligible influence on other elements [32]. In practice, rather than being physically removed, a “killed” element is retained with its stiffness matrix multiplied by a minimal factor (default 1.0 × 10^−6^). This reduction renders the element’s stiffness, load, mass, damping, and specific heat effectively zero, simulating its removal without altering the model topology. This approach enables the dynamic modification of element status during analysis, making it suitable for simulating material failure, damage, and progressive accumulation processes [33,34].

#### 2.1.3. Integrated Simulation Framework for FDM Temperature Field

**Figure 1:**
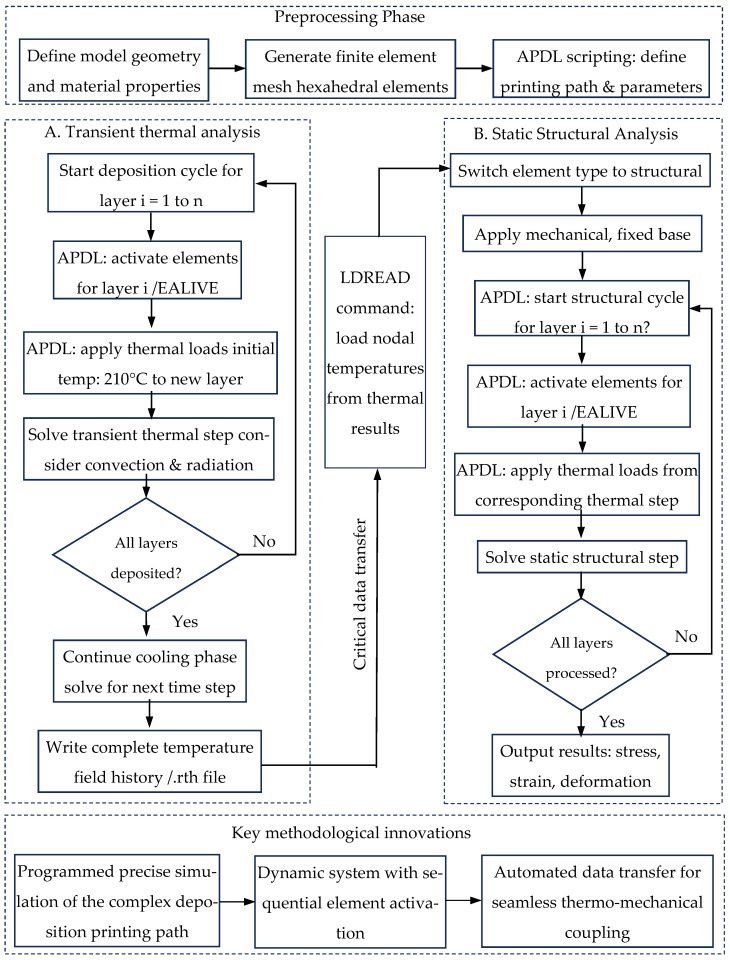
Flowchart of the sequentially coupled thermo-mechanical simulation framework for the FDM process, implemented via ANSYS APDL.

The core innovation of this work is the development and implementation of an integrated high-fidelity simulation framework that effectively combines the ANSYS APDL with the finite element technology. Their collaborative application can realistically simulate the FDM layer-by-layer printing process, from the initial deposition to the final cooling, the complete and path-related instantaneous thermal change process, which represents an important methodological advancement. However, the common commercial software methods usually rely on simplified assumptions or predefined geometric shape, making it difficult to accurately simulate the FDM printing process. The framework enables programmatic control of simulations, as depicted in Figure 1, and it has achieved three key innovations:

(1) Dynamic process replication: It allows for the precise, time-accurate activation of material elements (EALIVE command) in direct sequence with a user-defined, nonlinear printing path (linear reciprocating in this case), mirroring the actual manufacturing process rather than simulating a static, pre-deposited geometry.
(2) Automated multi-physics coupling: It automates the sequential thermo-mechanical analysis, ensuring perfect consistency in the history of deposition between the thermal and structural models. The critical data transfer of the temperature field is handled programmatically via the LDREAD command.
(3) Complex boundary condition management: It facilitates the application of complex, time-varying thermal boundary conditions (convection, radiation) on a dynamically changing model geometry, a task that is highly cumbersome and often approximated in standard graphical interface-based simulations.

### 2.2. A Sequentially Coupled Thermo-Mechanical Model for FDM Printing

The thermal process in FDM is governed by transient heat transfer with a moving heat source (the nozzle). This study employs the classical nonlinear transient heat conduction equation, which serves as the foundation for our simulation [35]. Its equation is shown in Formula (1).

(1) $$\rho c_{p}\frac{𝜕T}{𝜕t}=\frac{𝜕}{𝜕x}(k_{x}\frac{𝜕T}{𝜕x})+\frac{𝜕}{{𝜕}_{y}}(k_{y}\frac{𝜕T}{{𝜕}_{y}})+\frac{𝜕}{{𝜕}_{z}}(k_{z}\frac{𝜕T}{{𝜕}_{z}})+{\dot{q}}_{v}$$

where ρ represents density, $c_{p}$ is the temperature-dependent specific heat capacity, *T* is the temperature field, t is defined time, $k_{x},k_{y},k_{z}$ are the thermal conductivities, ${\dot{q}}_{v}$ is the volumetric heat source from the nozzle.

#### 2.2.1. Modeling of Latent Heat of Fusion

In this study, material modeling is the explicit and numerically stable incorporation of the latent heat of fusion for PLA. Instead of simply defining a temperature-dependent specific heat, we adopt the apparent heat capacity method within the framework of the enthalpy formulation. Total enthalpy (H) is defined as the integral of the apparent heat capacity. Its equation is as follows:

(2) $$H(T)=\int \begin{array}{c} T\\ T_{ref} \end{array}\rho c_{p,app}(T)dT$$

The apparent heat capacity ($c_{p,app}$) is constructed to smoothly encompass both the sensible heat and the latent heat (L) release/absorption over a temperature range around the melting point (*T_m_*), which can be determined as:

(3) $$c_{p,app}(T)=\left\{\begin{array}{cc} c_{p}^{solid}(T) & ,T<T_{m}+∆T\\ \frac{c_{p}^{solid}(T)+c_{p}^{liquid}(T)}{2}+\frac{L}{2∆T} & ,T_{m}-∆T\leq T\leq T_{m}+∆T\\ c_{p}^{liquid}(T) & ,T>T_{m}+∆T \end{array}\right.$$

Here, Δ*T* defines the phase transition temperature range. This formulation, implemented via a user-defined function in APDL, prevents numerical oscillations that plague simple step-function approaches and provides a more robust convergence for the highly nonlinear analysis.

#### 2.2.2. Sequentially Coupled Thermo-Mechanical Formulation

The thermal history drives the mechanical response. The governing equation for the static structural analysis is derived from the principle of virtual work, considering the thermal strains:

(4) $$\int_{v}\sigma :\delta \varepsilon dv=0$$

where *σ* is the Cauchy stress tensor and *δε* is the virtual strain tensor.

The key innovation in the coupling methodology is the decomposition of the total strain (*ε*) into mechanical and thermal components, with the thermal strain being a function of the simulated temperature history, which can be expressed as:

(5) $$\begin{array}{l} \varepsilon =\varepsilon_{mech}+\varepsilon_{th}\\ \varepsilon_{th}=\alpha (T-T_{ref})I \end{array}$$

where α is the coefficient of thermal expansion, *T_ref_* is the stress-free reference temperature (taken as the glass transition temperature), and *I* is the identity tensor.

The coupling is achieved programmatically: the nodal temperature results from the transient thermal analysis (*T* (*x*,*y*,*z*,*t*)) are read into the structural model as a body force load using the LDREAD command in APDL.

#### 2.2.3. Implementation of the Moving Heat Source

The nozzle is modeled as a moving volumetric heat source. Its spatial distribution at a given time is given by a Gaussian function centered on the nozzle path coordinates (*x*_0_(*t*), *y*_0_(*t*)), which can be expressed as:

(6) $${\dot{q}}_{v}(x,y,t)=\frac{p}{\pi r_{0}^{2}}\exp(-\frac{{\left(x-x_{0}(t)\right)}^{2}+{\left(y-y_{0}(t)\right)}^{2}}{r_{0}^{2}})$$

where *p* is the power of the heat source, and *r*_0_ is the characteristic radius of the nozzle’s thermal influence. This mode provides a more realistic representation of the heat input than a simple constant temperature boundary condition.

### 2.3. Material Model and Process Parameterization

PLA was selected as the model material in this work. Its properties are summarized in Table 1 [12]. The key material modeling refinement is the definition of specific heat capacity as a function of temperature to capture the phase change, while other properties were initially modeled as temperature-independent to establish a robust baseline. This focused approach allows for a clear isolation of the latent heat effect on the resulting temperature field and thermal stresses.

The process parameters, detailed in Table 2, were chosen to represent a standard industrial FDM setup [12,15]. In addition, the linear reciprocating scanning path was programmatically implemented in APDL.

During the thermomechanical coupled finite element analysis, the following assumptions and settings were applied to the numerical model:

(1) The PLA material is assumed to obey the von Mises yield criterion and the associated flow rule.
(2) The extruded filament is idealized as a perfectly flat strip, neglecting potential defects such as necking or fracture.
(3) Perfect contact is assumed between the newly deposited filament and the underlying layer, with heat transfer mechanisms including conduction, convection, and radiation.
(4) Heat dissipation during the forming process occurs solely through natural convection; the ambient temperature remains constant, and no additional external heat sources are considered.
(5) The effects of high-temperature material degradation and crystallization behavior are not accounted for in the model.

### 2.4. Finite Element Modeling and Boundary Setting

**Figure 2:**
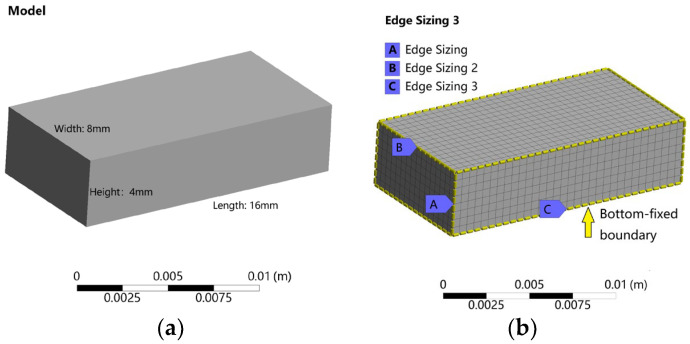
Finite element model setup and mesh configuration: (**a**) Geometry and dimensions of the cuboid model (4 mm × 8 mm × 16 mm); (**b**) Structured hexahedral mesh configuration (0.5 mm × 0.5 mm × 0.5 mm cells, 4096 elements).

In this study, a cuboid specimen measuring 4 mm × 8 mm × 16 mm was established as the computational domain, as shown in Figure 2a. The mesh design employed structured hexahedral elements with thermal coupling capabilities, selected for their stable spatial heat conduction and uniform heat flux transfer characteristics. A uniform element size of 0.5 mm was implemented throughout the computational domain, resulting in a total of 4096 equally sized elements. The corresponding structured hexahedral mesh is depicted in Figure 2b.

In addition, a bottom-fixed boundary condition was implemented to represent the constrained thermal deformation during printing, as illustrated in the figure. in Figure 2b. the Stefan-Boltzmann constant (σ = 5.67 × 10^−8^ W·m^−2^·K^−4^) and a convection coefficient of 72 W·m^−2^·°C^−1^ were set for all exposed surfaces. The total simulated time was 301.44 s, covering both the 101.44 s deposition and a subsequent 200 s cooling phase.

### 2.5. Numerical Implementation with APDL

The finite element analysis of the FDM process was implemented through ANSYS APDL to establish a high-fidelity computational framework capable of capturing the intricate thermo-mechanical phenomena inherent to the additive manufacturing process. This scripted methodology ensured precise control over the transient thermal and structural responses during the sequential deposition process.

#### 2.5.1. Element Birth–Death Activation and Path Control

The sequential material deposition was simulated utilizing the element birth-and-death capability. The complete geometry of the part was meshed prior to analysis initiation. All elements were initially “killed” (EKILL, ALL), scaling their stiffness and thermal conductivity matrices by a severe reduction factor to represent the pre-deposited state. The material addition was then accurately modeled by “reviving” elements (EALIVE) in a layer-by-layer, track-by-track sequence that mirrored the actual printing path. This was governed by nested loops controlling the deposition in the X, Y, and Z directions.

The fundamental algorithm for element activation and thermal initialization is demonstrated through the following critical command sequence:

(1) Sequential deposition loop.

1: *do, zcyc, 1, zlength/zinc, 1 ! Loop over Z layers

2: *do, ycyc, 1, ylength/yinc, 1 ! Loop along Y direction

3: *do, xcyc, 1, xlength/xinc, 1 ! Loop along X direction

(2) Select and activate elements in the current voxel.

1: **nsel**,s,loc,x,(xcyc-1)*xinc,xcyc*xinc ! X-path segment

2: **nsel**,r,loc,y,(ycyc-1)*yinc,ycyc*yinc ! Y-path segment

3: **nsel**,r,loc,z,(zcyc-1)*zinc,zcyc*zinc ! Current layer

4: **sln**,s,1 ! Select elements associated with the selected nodes

5: **esel**,u,**llive** ! Exclude already active elements

6: **ealive,all** ! Activate the selected (previously “killed”) elements.

(3) Apply Initial Thermal Condition.

1: **d,all,temp**,210 ! Apply deposition temperature (210 °C) to new elements.

This algorithmic implementation maintained both geometric and temporal accuracy in replicating the layer-wise material deposition process while ensuring computational efficiency through selective element manipulation. The temporal sequencing incorporated inter-layer cooling periods to accurately represent the actual manufacturing thermal cycle.

#### 2.5.2. Thermal Boundary Condition and Heat Exchange

Immediately after the activation of a new voxel, thermal boundary conditions were applied to model the heat exchange with the environment. The initial high temperature of the deposited material was defined, and convective cooling was applied to the top surface of the current layer.

The key commands for setting up the heat exchange are as follows:

(1) Convection on the top surface of the currently active layer.

1: **nsel**,s,loc,x,(xcyc-1)*xinc,xcyc*xinc

2: **nsel**,r,loc,y,(ycyc-1)*yinc,ycyc*yinc

3: **nsel**,r,loc,z,zcyc*zinc ! Select top face of current layer

4: **sf,all,conv**,72,25 ! Apply convection (h = 72 W/m^2^·K, T∞ = 25 °C)

5: alls

6: **solve** ! Solve transient thermal step.

(2) Remove fixed temperature to allow subsequent free thermal exchange.

1: **nsel**,s,loc,x,(xcyc-1)*xinc,xcyc*xinc

2: **nsel**,r,loc,y,(ycyc-1)*yinc,ycyc*yinc

3: **nsel**,r,loc,z,(zcyc-1)*zinc,zcyc*zinc

4: **ddele,all,temp** ! Allow subsequent free thermal exchange

5: alls.

This systematic approach to thermal boundary condition application enabled accurate simulation of the thermal history, capturing the essential physics of heat accumulation and dissipation throughout the deposition process. The convective boundary conditions effectively modeled the heat loss to the surrounding environment, while the sequential constraint management allowed for realistic thermal evolution of previously deposited material.

#### 2.5.3. Thermo-Mechanical Coupling Procedure

The thermal history from the transient analysis was directly used to drive a structural analysis to predict residual stress and deformation. A sequential coupling method was employed. First, a transient thermal analysis was conducted, followed by a static structural analysis. The same mesh and the same element generation sequence defined by the APDL loop were adopted to ensure that the evolution of the mechanical model matched the thermal history. The key coupling was achieved by importing the temperature field from the thermal analysis as a body load into the structural model using the “LDREAD” command.

The fundamental command structure for the structural solution is as follows:

1: **antype**, static ! Static structural analysis

2: **nropt**,full ! Full Newton-Raphson procedure

3: **ldread**, temp, ‘Thermal.rth’ ! Import temperature field from thermal analysis

solve.

In this procedure, the “LDREAD” command mapped the calculated nodal temperatures from the thermal results file onto the structural model as a body load. The resulting thermal strains, when constrained by the boundary conditions and the sequentially activated elements, generated simulated residual stress and distortion fields. The final state of stress and deformation after the complete cooling cycle, as analyzed in the results section, represents the simulated residual stress and warpage of the FDM part. This scripted methodology ensured a rigorous and reproducible simulation of the coupled thermo-mechanical phenomena governing the FDM process.

## 3. Results Analysis and Discussion

Based on the layer-by-layer deposition characteristics of the FDM process, this study simulates the thermo-mechanical coupling behavior during printing using ANSYS APDL with the element birth-and-death technique. By integrating heat diffusion and radiation principles, a custom APDL command flow was developed to control the simulation progression along the three coordinate directions of the model, accurately replicating the FDM printing path. The element birth-and-death method was employed to sequentially “activate” each deposited unit while keeping the remaining elements “inactive”, thereby realistically simulating the incremental buildup of the printed part. The transient temperature field distribution was obtained from the thermal analysis, which was subsequently used as the input for the structural analysis to compute the resulting stress and strain fields.

### 3.1. Temperature Distribution at Different Times During the Printing Process

**Figure 3:**
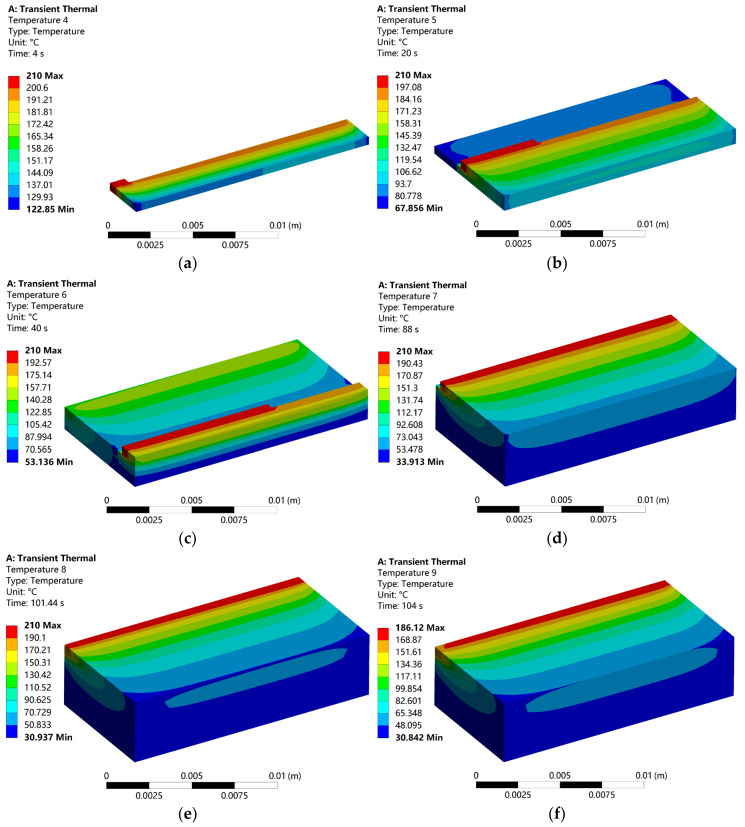
Dynamic temperature distribution at different times during the formation of FDM: (**a**) The time is 4 s; (**b**) The time is 20 s; (**c**) The time is 40 s; (**d**) The time is 88 s; (**e**) The time is 101.44 s; (**f**) The time is 104 s.

In this study, the temperature field during the FDM process was simulated and analyzed using ANSYS software (2023 R1). The total forming time was set as 101.44 s, followed by a cooling phase of 200 s. Using the post-processing functionality of the software, temperature distributions at different time steps were extracted, as shown in Figure 3.

The simulation results demonstrate that the predicted temperature evolution agrees well with the actual thermal behavior observed during the printing and layer-stacking process. After being melted at the maximum temperature of 210 °C, the filament is deposited and fused with previously formed material. During this stage, heat is transferred to the surrounding material and environment, followed by gradual diffusion and cooling to ambient temperature.

It is also observed that the temperature distribution exhibits clear spatial non-uniformity. Due to differences in heat dissipation conditions between the boundary and the interior of the part, heat diffuses more rapidly at the edges than in the central region. This non-uniform cooling can lead to localized thermal contraction and result in part deformation, thereby reducing dimensional accuracy. The simulation results clearly reflect this phenomenon, as the intermediate region retains heat for a longer duration compared to the periphery.

Figure 3a–f presents the temperature field nephograms at different stages of the FDM printing process, illustrating the dynamic thermal evolution during material deposition and cooling. As shown in Figure 3a–c, the temperature distribution exhibits a zonal gradient along the printing path. The energy input from the nozzle causes a sharp temperature to rise in the localized deposition zone, resulting in a high-temperature core region exceeding 200 °C and steep thermal gradients. The newly deposited material reaches the set nozzle temperature of 210 °C, while previously printed paths cool gradually, with temperatures dropping to approximately 53 °C within 40 s. Notably, even after 40 s, the initial layers have not yet equilibrated to the ambient temperature of 25 °C, indicating the persistence of residual heat in the part.

In Figure 3d, after an extended period of convective heat transfer with the ambient environment, the minimum recorded temperature is 33.91 °C, approaching but still above the room temperature setting. This is attributed to continuous heat diffusion from the upper, actively printed layers. Most of the structural elements remain around 100 °C, reflecting the material’s thermal retention. The internal regions exhibit slightly higher temperatures than the periphery, suggesting slower heat dissipation in the core, which may contribute to localized thermal stress accumulation. A significant temperature differential of up to 186 °C between the interior and exterior is observed, which could induce microcracks or interlayer debonding due to mismatched thermal contraction.

By the end of the printing process (Figure 3e), the final layer reaches 210 °C, while the minimum temperature is 30.94 °C. The outer surfaces cool rapidly due to high convective heat transfer with the ambient air, whereas the lower layers, having undergone prolonged cooling, approach the ambient temperature. The high-temperature region remains concentrated near the most recently deposited material.

Approximately 3 s after printing concludes (Figure 3f), the maximum temperature has declined to 186.1 °C, localized to the last printed track, while the minimum is 30.84 °C. This indicates ongoing heat diffusion, with the structure not yet fully stabilized to room temperature. The elevated temperatures around the final deposition path highlight the transient nature of heat transfer in FDM, consistent with nonlinear thermal behavior typical of additive manufacturing processes.

### 3.2. Temperature Distribution at Different Times During the Cooling Process

The FDM process encompasses two critical phases: the forming stage involving material deposition, and the subsequent cooling stage where complex thermo-mechanical phenomena occur. During cooling, the printed component undergoes continuous heat transfer with the surrounding environment through conduction, convection, and radiation mechanisms. The gradual dissipation of entrapped thermal energy leads the component toward thermal equilibrium with the ambient air. This transient thermal process is particularly crucial as spatial and temporal temperature variations induce thermal stresses that may compromise dimensional accuracy and structural integrity.

**Figure 4:**
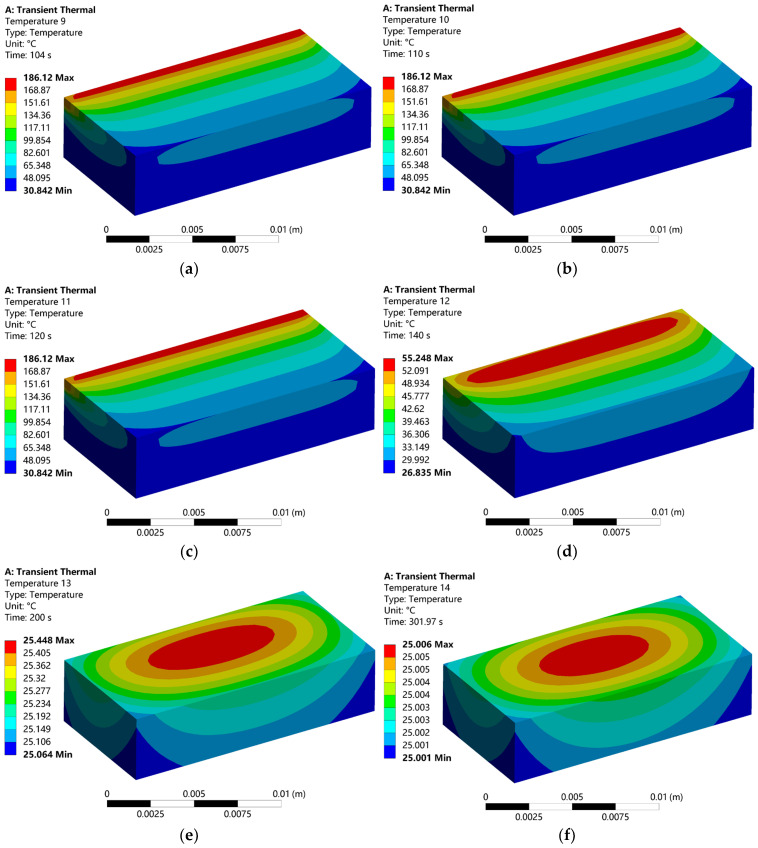
Dynamic temperature distribution at different times during FDM cooling process: (**a**) The time is 104 s; (**b**) The time is 110 s; (**c**) The time is 120 s; (**d**) The time is 140 s; (**e**) The time is 200 s; (**f**) The time is 301.97 s.

Figure 4 systematically presents the thermal evolution captured at six representative time intervals during the 200s cooling period. The initial cooling phase (104–140 s, Figure 4a–d) demonstrates a progressive temperature reduction from 186.1 °C to 55.25 °C at the maximum, while the minimum temperature increases from 30.84 °C to 26.84 °C, approaching the ambient temperature of 25 °C. The observed thermal distribution maintains a clear correlation with the deposition path, consistent with fundamental heat transfer principles as the thermal gradient gradually diminishes.

The persistent temperature elevation in central regions along the final deposition path (Figure 4d) reveals constrained heat dissipation characteristics in the part’s interior, contrasting with rapid cooling in peripheral areas exposed to enhanced convective heat transfer. This differential cooling behavior generates substantial thermal stresses through two primary mechanisms: first, the mismatch in thermal contraction between the solidified outer layers and the slowly contracting core establishes bending moments that directly contribute to warpage deformation; second, the high thermal gradients at interlayer boundaries induce shear stresses that may exceed the interdiffusion-based bond strength, potentially initiating delamination.

During advanced cooling stages (200–301.97 s, Figure 4e–f), the temperature field evolves into a concentric distribution pattern with maximum temperature (25.45 °C) localized in the geometric center and minimum temperature (25.06 °C) at the periphery. The minimal temperature differential of 0.39 °C confirms near-complete thermal stabilization, with Figure 4f demonstrating final achievement of thermal equilibrium at 25 °C. Critically, despite this uniform final temperature distribution, the residual thermal stresses generated during non-uniform cooling remain locked within the structure. These frozen-in stresses pose significant implications for dimensional stability, potentially manifesting as warpage during removal from the build platform or contributing to premature failure under service conditions.

The explicit relationship between observed temperature gradients and defect formation mechanisms underscores the importance of cooling rate control and thermal management strategies for optimizing FDM process parameters to minimize thermally induced defects and enhance final part quality.

### 3.3. Stress–Strain Analysis of FDM Process

During the FDM forming process, temperature variations exhibit nonlinear and transient characteristics. As heat diffuses and transfers within the printed part, complete and uniform cooling is seldom achieved due to ongoing thermal exchange between deposited layers. The cooling phase induces heterogeneous deformation across different regions of the part, primarily resulting from uneven temperature distribution. Specifically, the slower heat dissipation in the internal regions compared to the exterior leads to asynchronous cooling—where the outer layers solidify first while the inner sections remain at elevated temperatures. This differential cooling behavior generates localized stress concentration, particularly in the core region, and promotes noticeable deformation along the periphery, ultimately compromising the dimensional accuracy and overall forming quality of the fabricated part.

The intrinsic relationship between temperature variation and the resulting stress–strain response is governed by fundamental thermomechanical principles. During cooling, the material undergoes local contraction; however, this natural thermal shrinkage is constrained by the non-uniform temperature field and the mechanical confinement imposed by already solidified layers. This constraint mechanism directly generates thermal stress, the magnitude of which is proportional to the local temperature change and the material’s thermomechanical properties. The associated thermal strain, manifesting as deformation, is driven by the same thermal mismatch. Consequently, regions experiencing steeper thermal gradients or higher cooling rates develop greater internal stresses, leading to the heterogeneous strain distribution observed in the final part.

**Figure 5:**
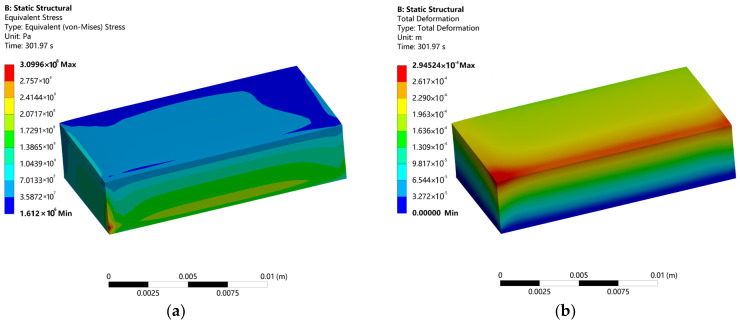
FDM forming stress–strain field: (**a**) Stress distribution; (**b**) Strain distribution.

Figure 5 illustrates the thermal stress and strain distributions developed during the FDM forming and cooling stages. As shown in Figure 5a, the maximum equivalent von-Mises stress reaches approximately 310 MPa, located predominantly near the bottom region of the part. This value exceeds the yield strength of typical PLA material by a factor of about five, indicating the likelihood of irreversible plastic deformation. The stress distribution decays gradually along the build direction, decreasing from nearly 200 MPa at the bottom to about 70 MPa at the top—a trend that correlates well with the thermal gradient, thereby confirming the presence of strong thermomechanical coupling. Within individual layers, the central zones experience higher stress levels than the peripheral areas, with more pronounced stress variations observed in upper layers. This non-uniform stress state promotes an outward expansion tendency, resulting in localized distortions such as edge warping and center buckling.

As depicted in Figure 5b, the total deformation distribution exhibits similar spatial heterogeneity. The maximum displacement of approximately 2.95 × 10^−4^ m occurs on the upper surface, while the minimum value is 3.68 × 10^−5^ m at the bottom. A monotonic increase in deformation is observed from the base to the top surface, following a layer wise progression consistent with the distribution of both temperature and thermal stress. This pattern reflects the underlying deformation mechanism: the central region accumulates greater strain energy relative to the periphery. Such strain localization not only contributes to warpage but may also lead to residual stress concentrations in the core zone, ultimately compromising the mechanical integrity of the printed component.

## 4. Experimental Verification

To validate the accuracy and reliability of the finite element simulation framework established in this study, a series of experimental measurements were conducted to compare with the simulation results in terms of temperature distribution and stress-strain behavior.

### 4.1. Experimental Setup and Procedure

A desktop FDM 3D printer was used for experimental validation. The printing material was PLA filament with a diameter of 1.75 mm, consistent with the simulation. The printed part geometry was a cuboid of 4 mm × 8 mm × 16 mm, matching the simulation model. The process parameters, including nozzle temperature of 210 °C, print speed of 80 mm/s, and layer height of 0.2 mm, were strictly aligned with the settings in Table 2.

This study developed an experimental system capable of simultaneous temperature monitoring and stress measurement. For strain measurement, the primary sensor employed is the TSK-89561H strain gauge, which maintains stable operation over a temperature range of −30 °C to 250 °C with a measurement error of <1%. Fabricated with a glass fiber-reinforced polyimide substrate and Kapton foil, its detailed specifications are presented in Table 3. This strain gauge exhibits excellent thermal stability and reliable electrical insulation, rendering it particularly suitable for stress analysis in thermally dynamic environments.

In addition, a platinum resistance temperature sensor (PRT-PT100; PRT: Platinum Resistance Thermometer) was used to acquire temperature variation data during the printing process. This sensor spans a temperature range of −200 °C to +600 °C with an accuracy of 0.15 °C and features a thin-film structure with a compact 2 mm form factor. Its detailed specifications are presented in Table 4. Leveraging its compact design, the sensor can be easily integrated into the interior of printed components, thereby fully satisfying the temperature variation measurement requirements for printed parts in this study.

**Figure 6:**
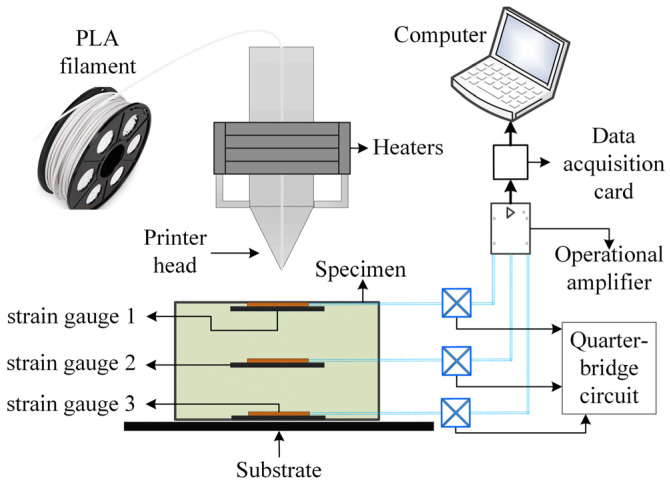
Experimental schematic diagram for temperature and strain measurement.

The schematic of the experimental testing principle for this study is illustrated in Figure 6. In this experiment, sensor mounting slots were pre-reserved at three key locations of the printed component: the bottom, middle layer, and top surface center. During strain measurement, the printing process was temporarily paused each time the printer head reached one of the pre-reserved slots. A strain gauge was then bonded to the corresponding slot using high-temperature-resistant adhesive, after which printing resumed. To minimize measurement errors, the strain gauge was connected in a three-wire configuration and a quarter-bridge circuit. The voltage signals generated by the strain gauge were amplified, recorded via a data acquisition (DAQ) card, and subjected to subsequent data processing—enabling quantitative determination of the component’s deformation behavior. For temperature measurement, a separate printing trial was conducted. Three temperature sensors were installed at the same three reserved slots, and its output signals were directly amplified before being collected by the DAQ card. Post-processing of the acquired temperature data allowed the characterization of temperature variations in the component throughout the printing process.

**Figure 7:**
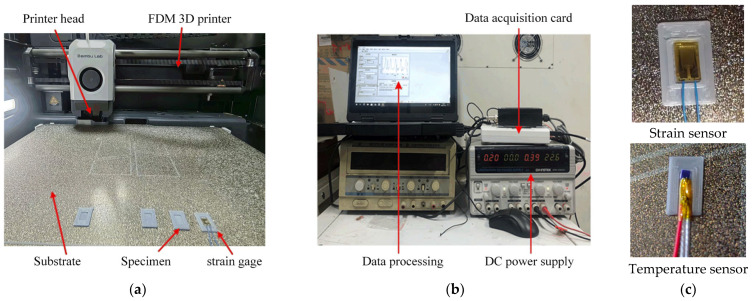
FDM printing process temperature and strain measurement experimental system: (**a**) 3D printer; (**b**) Data acquisition; (**c**) Sensors.

An experimental system for measuring temperature and strain during the FDM printing process was set up in the laboratory, as shown in Figure 7. It mainly includes FDM 3D printers, measurement sensors, and data acquisition and processing systems.

### 4.2. Comparison of Temperature Field

**Figure 8:**
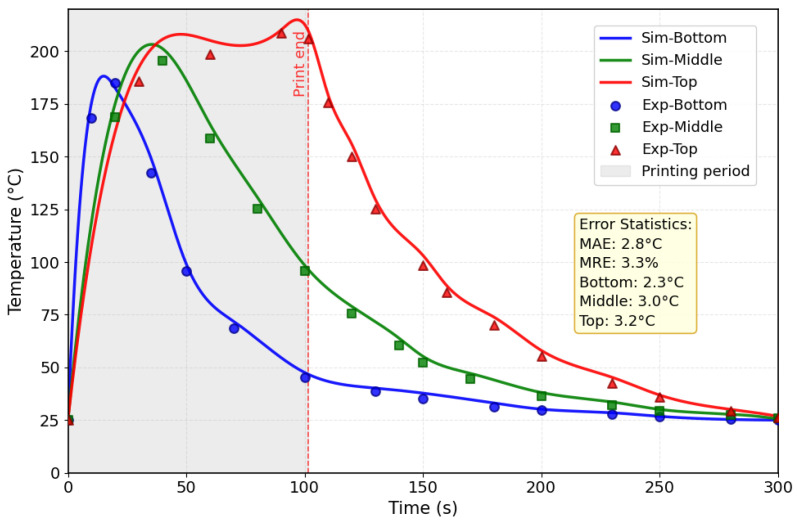
Comparison of experimental and simulated temperature changes curves at the three different locations.

Based on the comparative analysis of the internal temperature profiles obtained from strain gauges measurements and numerical simulations at corresponding locations (Figure 8), a strong agreement between the simulated and experimental results is observed throughout the process. The model accurately captures the characteristic thermal cycles, including the rapid temperature rise during material deposition and the subsequent cooling phase. Quantitative evaluation reveals that the maximum temperature deviation between simulation and experiment remains below 8%, while the overall Mean Absolute Error (MAE) and Mean Relative Error (MRE) across all measurement points are 2.8 °C and 3.3%, respectively.

Further analysis of local discrepancies shows location-specific MAE values of 2.3 °C at the bottom, 3.0 °C at the middle, and 3.2 °C at the top regions, indicating slightly increased deviation at higher locations. These minor variations fall well within acceptable bounds for such complex thermal processes. The consistent correlation between simulated and experimental data across all monitored locations confirms that the APDL-based finite element model, incorporating the birth-and-death element technique and latent heat effects, provides a reliable prediction of the transient thermal behavior inherent to the Fused Deposition Modeling process. The model’s accuracy in replicating both the magnitude and trend of temperature evolution demonstrate its effectiveness as a robust computational tool for analyzing the thermal history in FDM.

### 4.3. Validation of Stress and Strain Distribution

**Figure 9:**
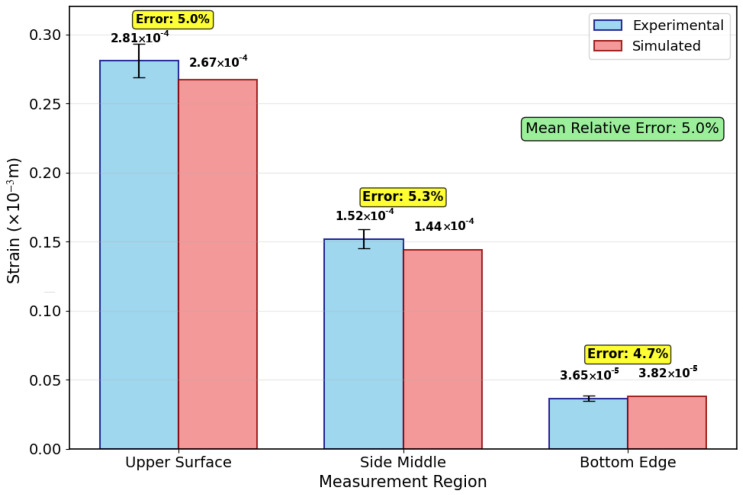
Comparison of experimental and simulated strain changes curves at the three different locations.

Figure 9 demonstrates strong agreement between experimental and simulated strain distributions across all measured regions. The strain decreases progressively from the upper surface (2.81 × 10^−4^ m) to the bottom edge (3.65 × 10^−5^ m), following the thermal gradient pattern observed during FDM processing. Simulation accuracy is evidenced by low relative errors of 5.0%, 5.3%, and 4.7% for upper, middle, and bottom regions, respectively, with a mean error of 5.0%.

This strain distribution results from differential thermal contraction constrained by the build platform and previously deposited layers. The upper surfaces experience less constraint, allowing greater deformation, while bottom regions remain more restricted.

Although direct residual stress measurement remains challenging, the close correlation between predicted and observed strain distributions (mean error: 5.0%) provides robust indirect validation of the stress predictions in Figure 5a, confirming the reliability of the thermomechanical coupling analysis.

### 4.4. Model Validation and Limitations

The comprehensive validation presented in this study demonstrates the robust performance of the integrated APDL and birth–death element approach in simulating both thermal and mechanical aspects of the FDM process. The close agreement between experimental and numerical results, with average errors below 5% for both temperature and strain predictions, underscores the methodology’s effectiveness in capturing the complex Multiphysics phenomena involved in additive manufacturing. Minor deviations between the simulated and experimental temperatures are attributed to factors not fully captured in the model, such as minor fluctuations in ambient airflow, slight variations in material properties, and the idealized assumption of the heat source model.

Nevertheless, the overall high level of agreement between simulation and experiment demonstrates that the proposed methodology provides a credible and effective tool for predicting the thermo-mechanical phenomena in FDM, offering valuable guidance for practical process optimization and quality control.

## 5. Conclusions

This study establishes a robust finite element framework integrating ANSYS APDL with birth–death element technology for simulating coupled thermomechanical behavior in FDM processes. The main findings are:

(1) The integrated APDL and birth–death element approach effectively simulates the complete FDM thermal cycle, including dynamic material deposition and cooling phases. Experimental validation confirms the model’s accuracy, with temperature prediction errors below 8% and strain distribution errors within 5%.

(2) Temperature distribution exhibits path-dependent gradient characteristics, with rapid surface cooling and significant heat accumulation in internal regions due to PLA’s low thermal conductivity. This inhomogeneous cooling induces substantial thermal stress up to 310 MPa at the bottom part.

(3) Thermomechanical analysis reveals distinct stress–strain patterns: maximum stress concentration occurs at the constrained bottom region, while strain increases progressively toward the upper surfaces (from 3.68 × 10^−5^ m to 2.95 × 10^−4^ m), corresponding to the observed warpage deformation.

The proposed methodology provides a reliable computational tool for predicting thermal-induced deformations in FDM processes, offering valuable insights for process parameter optimization and quality control in industrial applications.

## Figures and Tables

**Table 1:** Polylactic acid material parameters.

Density (kg/m^3^)	Thermal Conductivity (W/m·°C)	Elastic Modulus (MPa)	Poisson’s Ratio	Specific Heat Capacity (J/kg·°C)	Temperature (°C)
1250	0.25	3.5 × 10^3^	0.35	1560	47.5
				1700	54.9
				1820	60.3
				1900	109.3
				2320	134.9
				4360	145.6
				2100	152.0
				1980	172.3

**Table 2:** Printing process parameter setting for finite element simulation.

ConsumaBles	Fill Rate	Layer Height	Print Temperature	Print Speed	Print Spacing	Print Trajectory
PLA	30%	0.2 mm	210 °C	80 mm/s	0.4 mm	Linear reciprocating scanning

**Table 3:** Strain gauge technical parameters.

Strain Gauge	Strain Grid	Resistance	Temperature Range	Resolution	Error
TSK-89561H	0.7 mm	120 Ω	−30–250 °C	1 με	<1%

**Table 4:** Platinum resistance thermometer technical parameters.

Platinum Resistance Thermometer	Structural Type	Temperature Measurement Range	Accuracy	Error
PRT-PT100	Thin-Film Type	−200–+600 °C	High	<±0.15 °C

## Data Availability

Data will be provided on request due to privacy through the corresponding author (Yuehua Mi) of this article.
